# COVID-19 and the Curse of Piecemeal Perspectives

**DOI:** 10.3389/fvets.2020.582983

**Published:** 2020-09-23

**Authors:** Chris Walzer

**Affiliations:** ^1^Health Program, Wildlife Conservation Society, Bronx, NY, United States; ^2^Conservation Medicine Unit, Research Institute of Wildlife Ecology, Department of Interdisciplinary Life Sciences, University of Veterinary Medicine, Vienna, Austria

**Keywords:** COVID-19, SARS-CoV-2, wildlife, trade, market

## Abstract

The world is in turmoil. A novel coronavirus (SARS-CoV-2) has catapulted across the ever-evolving interface between humans and wild places relentlessly spreading coronavirus disease (COVID-19) amongst humans and bringing immense suffering and death to the farthest reaches of our planet. What was immediately apparent was that the virus responsible for this outbreak originated in wild animals. A wildlife source does not come as a surprise as the majority of emerging infectious diseases are zoonotic and two-thirds have their origin in wildlife. The commercial use of wildlife for consumption encompassing both legal and illegal trade is poorly regulated with porous boundaries between the two entities. This trade, particularly in live animals, creates super-interfaces along the food value chain co-mingling species from many different geographies and habitats while creating perfect conditions for the exchange and recombination of viruses. Since the SARS outbreak in 2002/2003, broad scientific consensus exists that long term, structural changes, and wildlife trade and market closures will be required to prevent future epidemics. The pragmatic, most cost-effective action governments can take with immediate effect is to ban the commercial trade of wild birds and mammals for consumption. Most importantly, this reduces the risk of future zoonotic transmission while also safeguarding resources for those Indigenous Peoples and local communities who rely on wild meat to meet their nutritional requirements.

## Introduction

The world is in turmoil. A novel coronavirus (SARS-CoV-2) has catapulted across the ever-evolving interface between humans and wildlife relentlessly spreading coronavirus disease (COVID-19) amongst humans and bringing immense suffering and death to the farthest reaches of our planet. Quarantines have been imposed; borders have been closed. Free movement of people and the pursuit of normal daily routines have been dramatically curtailed by a virus that previously existed beyond the pale and a disease that was unknown and unnamed only a few months ago. What was immediately apparent was that the virus responsible for this outbreak originated in wild animals (1). A wildlife source does not come as a surprise as the majority of emerging infectious diseases are zoonotic. Globally, more than 335 Emerging Infectious Disease (EID) outbreaks, involving 183 distinct pathogens, were reported between 1940 and 2004 (2). That's more than 50 outbreaks per decade, and the rate is increasing. More than half (52%) of all EID events in recent years originated in wildlife (2). Among emerging zoonoses specifically, 72% of outbreaks have originated in wildlife with the rest emerging from domestic animals (2). Emerging zoonoses have significant implications for both public health and economic stability with the costs of many individual recent major outbreaks such as SARS, MERS and Ebola estimated in the tens of billions of US dollars. These costs exceed 1–2% of GDP in less wealthy countries and surpass the International Monetary Fund's threshold (0.5% GDP loss) for major economic disasters (3). When all is tallied, it is certain that the economic devastation caused by COVID-19 will be orders of magnitude greater: in the trillions to tens of trillions of US dollars.

## What Do We Know?

As in previous zoonotic coronavirus spillover events of global concern, a bat species is most likely the evolutionary host to the on-going SARS-CoV-2 pandemic (4). Initially, in December 2019, human cases, were epidemiologically linked to a seafood market in Wuhan, China, where live wild animals were sold and slaughtered for consumption (5). However, not all of the first human cases were market associated. To date, timing, location and mechanisms of the spillover event(s) have not been conclusively determined and possibly will never be due to the apparent lack of animal sampling in the early days of the outbreak (5). All three zoonotic-origin coronaviruses (SARS-CoV, MERS-CoV, and SARS-CoV-2) result from recombination. Ancestral and recent viral recombination events between bats, pangolins, and still to-be-identified additional hosts most likely made it possible for SARS-CoV-2 to acquire the attributes necessary to infect human cells and subsequently transmit from humans to humans (6).

## The Wildlife Trade for Consumption

While robust data is lacking, the legal and illegal trade in wildlife is valued at hundreds of billions in US dollars (7). Wildlife trade is driving species extinctions and is a critical factor in global biodiversity loss (8). The illegal trade in wildlife is the fourth most profitable crime after drugs, human trafficking, and arms and generates at least USD 23 billion in illicit annual revenue (9). Data on the value of the global commercial wildlife trade for consumption is sparse, but the global total annual value of wildlife harvesting is estimated at USD 400 billion (10). This sum includes household community-based hunting for subsistence consumption and surplus sale, but a far greater proportion reflects community-external hunting that supply national and international trade (10). It is thought that there are some 20,000 wildlife farms, employing more than 6 million people and generating an estimated USD 18 billion dollars in China alone (11). Across southern Viet Nam, 4,099 active farming operations, stocking an estimated one million wild animals (including, rodents, primates, civets, wild boar, Oriental rat-snakes, deer, crocodiles, and softshell turtles). were recorded (12). These farming operations supply wild animals predominantly for meat for human consumption and sell to national urban wild meat restaurants that serve increasingly affluent populations. They simultaneously supply international markets with wild meat (13). The commercial use of wildlife for consumption encompasses both legal and illegal trade that is poorly regulated with porous boundaries between the two entities [e.g., (14)]. The trade involves the capture, transport, and containment of wild animals. These activities induce stress, injury, sickness, and compromise immune systems. The multiple stressors inhibit animal immune responses and allow for enhanced shedding of pathogens (15). Stress also leads to increased excretion of saliva and voiding of urine and feces, all of which facilitate the shedding of viruses.

Genetic change in viruses is driven by several mechanisms, amongst them recombination, which occurs when two or more viral genomes co-infect the same host cell and can exchange genetic segments (16). This trade, particularly in live animals, creates super-interfaces along the food value chain co-mingling species from many different geographies and habitats (that would never have otherwise come into contact). A recent study from Vietnam demonstrated that the odds of coronavirus RNA detection among field rats (*Rattus* sp. and *Bandicota* sp.) destined for consumption increased significantly along the supply chain from traders to markets to restaurants (17). Wildlife trading sites, as in the Wuhan market, are vast, industrialized centers, cramming thousands of live animals from hundreds of species alongside thousands of domestic animals. This contrasts starkly with small stalls where local communities exchange and sell wildlife for subsistence. Furthermore, not only do animals exchange viruses among themselves, but vendors and customers also circulate within this milieu while slaughter and purchasing practices continually generate potential spillover opportunities. The commercial live wildlife trade and wildlife markets constitute true caldrons of contagion.

## What Needs to be Done in the Future?

First and foremost, we must acknowledge the basic tenet addressed by World Health Organization (WHO) Director-General Dr. Tedros Adhanom Ghebreyesus: “*The pandemic is a reminder of the intimate and delicate relationship between people and planet. Any efforts to make our world safer are doomed to fail unless they address the critical interface between people and pathogens, and the existential threat of climate change, that is making our Earth less habitable”*(18). We also have to acknowledge that zoonotic spillover events and subsequent outbreaks are inevitable, as the interfaces between wildlife and humans increase, primarily due to deforestation and agricultural expansion (19). However, our collective and determined actions can prevent outbreaks from becoming global pandemics. Reducing spillover opportunities necessitates multi-faceted approaches that include amongst others, considering wildlife pathogen impacts during land-use change, social marketing campaigns to reduce wildlife demand, providing alternative protein and micro-nutrient sources, strengthen law enforcement response to illegal wildlife trade. While much insight has been gained in the past decade, in part due to large research consortiums such as the USAID-funded PREDICT projects, there are still substantial gaps in knowledge concerning, amongst others, viral threats and spillover mechanisms. Future multidisciplinary and well-funded collaborative One Health approaches are urgently needed to quantify and prioritize spillover risks while informing decision-makers on implementing risk reduction measures. Pre-emergence research and surveillance need to be paired with participatory, just and community-informed social and behavioral change measures and global outbreak preparedness capacity strengthening.

## What Needs to be Done Now?

The pragmatic, most cost-effective action governments can take with immediate effect is to ban the commercial trade of wild birds and mammals for consumption. Most importantly, this significantly reduces the risk of future zoonotic transmission while also safeguarding resources for those Indigenous Peoples and local communities (IPLCs) who rely on such. Furthermore, it protects global biodiversity (20). This expedient and straightforward risk mitigation measure is surprisingly contentious in the public arena. Four unsound and inconsistent approaches are presently being widely promoted in the media, and to governments and donor institutions: (i) The sole focus on markets is inherently flawed as markets constitute just one part of the wildlife trade supply chain. Along the supply chain, multiple points pose a high risk of zoonotic pathogen transmission, including wholesale trader warehouses, stores, transport, wildlife farms, restaurants, pet shops, and border crossing points where wildlife is consolidated (18, 21); (ii) Similarly, vocal advocacy for closure of only the (as yet undefined) 50 highest-risk markets represents a dangerously unsound approach (22) that discounts the magnitude of the problem: Following China's Standing Committee of the National People's Congress decision to eliminate the consumption of wild animals for food to safeguard people's lives and health on the 24 February 2020, the National Forestry and Grassland Administration confiscated 39,000 wild animals and “cleaned up” more than 350,000 sites, such as restaurants and markets where wildlife was traded. Additionally, some 17,000 online accounts and e-commerce platforms trading wildlife products were closed down. Closing 50 markets appears frivolous at best (23); (iii) The focus on so-called high-risk species lacks evidence and defies enforcement. Numerically abundant orders such as rodents and bats harbor more viruses, but the notion of “special viral reservoirs” has recently been revoked (24). Most pathogens in wildlife remain unidentified, and many spillover events are overlooked (19). Less than 300 viruses from 25 high-risk viral families in mammals and birds are known to infect people. Yet, it is estimated that there are around 1.7 million viruses from these same viral families that have not yet been discovered. About 700,000 are predicted to have zoonotic potential (25); (iv) Enforcing hygienic standards, sanitizing markets and restaurants that sell wildlife is similarly being heavily promoted by numerous wildlife trade-related organizations (22, 26). There is ample evidence, especially from the avian influenza literature, that hygiene and management measures cannot prevent the resurgence of outbreaks (27).

## Discussion

Since the SARS outbreak in 2002/2003, broad scientific consensus exists that long term, structural changes, and wildlife trade and market closures will be required to prevent future epidemics (6, 28, 29). This mode of action is now also supported by intergovernmental organizations, such as the WHO, and international legal instruments, such as the Convention on Biological Diversity (29). In contrast to what some authors have suggested, no one, to my knowledge, is under the impression that closing down the global commercial trade of wildlife for human consumption is simple or that this is the only measure that needs to be addressed. Playing one necessary measure against another, confusing Central Africa with the situation in South-East Asia and China is simplistic and negligent (30). Based on the robust scientific evidence available, we must stridently reject assertions that cultural importance and the economic value of commercial wildlife meat retail, outweigh a devastating global pandemic that has impacted the entire planet, caused hundreds of thousands of deaths and cost the global economy USD trillions. Ostensibly raising concern for food security of IPLCs is a thinly veiled smokescreen to enable a return to business as normal while distracting from the fact that large, live-wildlife-trading markets in South-East Asia and China predominantly cater to the economically empowered middle and upper classes supplying expensive wild luxury meats and ego-bolstering status symbols. Food security and rights of IPLCs do not rely on international trade in live wildlife. On the contrary, this unsustainable, profit-oriented trade empties the forests of the very wildlife the IPLCs depend on (31). Furthermore, it has been estimated that the COVID-19 pandemic will add somewhere between 83-132 million people to the total number of undernourished people on this planet (32). Most importantly, rejecting scientific evidence paired with unclear and myopic messaging undermines the progress being made in key wildlife trade countries. In China, law-makers in the National People's Congress are moving toward legislating the February Decision to prohibit the trade of wild animals for human consumption. In Vietnam, following the announcement in March 2020 by the Prime Minister Nguyen Xuan Phuc to “take strong and sustainable actions to halt all illegal wildlife trade and consumption in Vietnam,” a new taskforce committed to reforming policies to prohibit the commercial trade and consumption of wild birds and mammals has launched into action. While these legislative actions are to be commended, it is essential to pair these with pervasive educational and social marketing measures to drive change across civil societies concerning wildlife usage. For preventive measures to persist in the long term, global funding support is required. Recently, these global preventive costs for 10 years are estimated at 2% of the costs of the COVID-19 pandemic (33).

The increasing incidence of viral spillover events is a symptom of ailing planetary health. As human activities and encroachment increasingly undermine the integrity of naturally balanced ecosystems, environmental health, and resilience are compromised affecting all species on the planet. Spillover events reflect impact not just on human health but the health of all the earth's organisms. Viral, species switching, and spillover events into humans are simple. It all comes down to a numbers game: the more often we force conditions that drive increases in direct contacts of wildlife and humans, the higher the likelihood of another spillover event. Timidly tackling a limited number of markets and developing standards that purportedly regulate and sanitize wildlife trade are backward-looking reductionist approaches based on naïve simplifications of interdependencies in disease emergence, economic development, and global interconnectedness.

The time has come for the global community to collectively assume responsibility for the negative externalities of the commercial trade in wildlife for consumption. The world has irrevocably changed and there can be no going back. As we, the global community, strive to build back better, we must ensure that future food production and security is healthy, sustainable and supports planetary health. A transition of global food production from being a major part of the health, climate and biodiversity crisis toward food production playing a central part in the solutions. We need bold, forward-reasoning organizations and leaders who acknowledge root causes, take responsibility and weather the inevitable pushback from narrowly focused interest groups while also overcoming traditional economic and disciplinary silos to design future health and well-being for all.

## Data Availability Statement

The original contributions presented in the study are included in the article/supplementary material, further inquiries can be directed to the corresponding author/s.

## Author Contributions

CW conceived, developed, researched, and wrote the manuscript.

## Conflict of Interest

The author declares that the research was conducted in the absence of any commercial or financial relationships that could be construed as a potential conflict of interest.
